# Profile of Volatile Compounds in Dessert Yogurts Prepared from Cow and Goat Milk, Using Different Starter Cultures and Probiotics

**DOI:** 10.3390/foods10123153

**Published:** 2021-12-20

**Authors:** Georgia Papaioannou, Ioanna Kosma, Anastasia V. Badeka, Michael G. Kontominas

**Affiliations:** Laboratory of Food Chemistry, Department of Chemistry, University of Ioannina, 45110 Ioannina, Greece; g.papaioannou@uoi.gr (G.P.); i.kosma@uoi.gr (I.K.); abadeka@uoi.gr (A.V.B.)

**Keywords:** dessert yogurt, cow milk, goat milk, starter cultures, probiotics, volatile compounds, organoleptic evaluation

## Abstract

The purpose of this study was to determine the profile of volatile compounds (aroma) and overall flavor in dessert yogurts prepared from cow and goat milk, using three different, commercially available starter cultures, in the presence or absence of probiotic bacteria and to correlate this to organoleptic evaluation results obtained using a consumer acceptability method. The extraction of volatile compounds was carried out by solid phase micro-extraction; separation and analysis by gas chromatography-mass spectrometry. Variations among the different classes of compounds (i.e., aldehydes, alcohols, ketones, volatile acids, hydrocarbons, and terpenes) were recorded for different treatments. The results showed that the main volatiles in the cow milk dessert yogurts without *Bifidobacterium* BB-12 were: acetaldehyde, 2,3-butanedione, 2,3-pentanedione, 3-OH-2-butanone, 2-propanone, hexanoic acid and limonene). Respective volatiles in cow milk dessert yogurts with *Bifidobacterium* BB-12 were: acetaldehyde, pentanal, hexanal, the same ketones, acetic acid and limonene). The volatiles in goat milk dessert yogurts without *Lactobacillus acidophilus* LA-5 were: acetaldehyde, the same ketones, no carboxylic acids, limonene, camphene, α- and β-pinene. Respective volatiles in goat milk dessert yogurts with *Lactobacillus acidophilus* LA-5 were: aldehydes acetaldehyde, the same ketones, butanoic acid, α-pinene and camphene varying in concentration in different samples. Based on the results of volatiles and organoleptic evaluation, it can be concluded that dessert yogurts from cow milk without probiotic bacterial strains using the mild and classic starter cultures, and dessert yogurts from goat milk with probiotic bacterial strains using the classic and acidic starter cultures are found to be more organoleptically acceptable by consumers. In most cases, a positive correlation was found between dessert yogurt organoleptically determined flavor and volatiles (aldehydes, ketones and carboxylic acids).

## 1. Introduction

Milk is a food produced by mammals to feed their offspring. Due to its high nutritional value and its good flavor, milk has been one of the main components of human nutrition since ancient times [1]. Aside from its direct consumption, milk has been used to produce a wide range of dairy products. These products are usually prepared by fermentation with specific microorganisms. The variety of these products, especially in recent years, is large (sour milk, yogurt, cheese, butter, etc.) [2,3]. One of the most representative dairy products around the world is yogurt [2]. Yogurt is a very nutritious and easily digestible food that is suitable for all ages, making it one of the most frequently consumed foods [3]. In addition to the above, the popularity of yogurt among consumers depends mainly on its sensory characteristics, of which aroma, taste and texture are most important.

In fact, the properties of foods that can be perceived by the senses (e.g., color, texture, flavor) influence the choice of the product by consumers more than its nutritional value [4]. For example, a food may have an extremely high nutritional value, but its appearance may not be attractive to consumers, or its taste and aroma may prevent consumers from buying it [5]. Therefore, the sensory properties of foods greatly contribute to product quality and acceptability by consumers.

Volatile compounds are responsible for the aroma of foods. As aroma is critical for the acceptance of a food, it plays an important role in its commercialization. For this purpose, various studies have been carried out in order to identify the volatile compounds of foods including yogurt [6,7,8,9,10,11,12,13,14,15]. Of course, a number of volatile compounds are not always perceived as characteristic odors, as the compound contributing to a particular odor should be at a concentration greater than a minimum threshold [7]. It should be noted, however, that most odors are not attributed solely on a single volatile component, but to a mixture of compounds, some of which may be present at sub-threshold levels.

In addition, it has been observed that in yogurts deriving from milk of different animal origin (i.e., cow vs. goat vs. ewe), the profile of the volatile compounds differs significantly, giving yogurts different organoleptic properties. Aside from the above, the fermentation process and other phenomena such as the oxidation of lipids contribute specific aromatic notes. As such, the aroma of the dessert yogurts is also affected by the type of microorganism strain used for the fermentation of the milk. More specifically, the formation of VOCs in fermented dairy products is complex and includes the glycolysis, lipolysis as well as the proteolysis of milk components, which is brought about by the enzymatic mechanisms of microflora within the dairy matrix [16].

Finally, in order to achieve optimum health benefits for the consumer, probiotics are usually added to yogurts which, in turn may affect product sensory properties. Based on the above, the objective of the present study was to determine the effect of (i) milk type (cow vs. goat), (ii) starter culture (sweet-mild, classic and acidic) and (iii) type of probiotic culture (*Bifidobacterium* BB-12 in cow milk and *Lactobacillus acidophilus* LA-5 in goat milk) on the aroma and the flavor (aroma + taste) of dessert yogurt. To the best of our knowledge, the above-mentioned experimental parameters have not been previously studied simultaneously in yogurts or dessert yogurts. The present study attempts to illustrate that an optimum selection with regard to starter culture and probiotic culture may result in a dessert yogurt (cow or goat) with the most desirable aroma profile and overall consumer acceptability.

## 2. Materials and Methods

### 2.1. Dessert Yogurt Dessert Preparation

In order to prepare, the set type, dessert yogurts, whole cow milk (fat: 3.8–4.0%) and goat milk (fat: 4.3–4.6%) were used. Milk was donated from DODONI SA dairy Co. (Ioannina, Greece). Protein concentrate was used to improve the structure of the dessert yogurts. The protein concentrate (SA-Nutrilac^®^ Yo 7700) was purchased from Arla Foods Hellas -Athens, Greece) and prepared using cow milk. The protein concentrate from cow milk consisted of 75–79% protein, 7–10% lactose, fat < 5%, ash < 7% and <6% moisture. For the fermentation of milk, three thermophilic lyophilized Direct Vat Set (DVS) cultures were used. The cultures, sweet-Mild (YoFlex^®^ Mild 1.0), Classic (YF-L812), and Acidic (YF-L903) consisted of strains of *Lactobacillus delbrueckii* subsp. *bulgaricus* and *Streptococcus thermophilus* and were purchased from Chr. Hansen (Hoersholm, Denmark). 

The probiotic cultures used were a thermophilic culture of *Bifidobacterium* (nu-trish^®^ BB-12^®^) for cow milk and a thermophilic culture of *Lactobacillus acidophilus* (nu-trish^®^ LA-5^®^) for goat milk, purchased from Chr. Hansen (Hoersholm, Denmark). In the case of goat yogurt, probiotics were also used to mask the unpleasant odor and taste of the fermented product deriving from goat milk [17]. *Lactobacillus acidophilus* LA-5 was specifically chosen as a probiotic culture in goat yogurt based on various studies ([18]) reporting the enhancement of the sensory acceptability of goat milk yogurt using this probiotic culture.

First, to prepare the dessert yogurts, the protein concentrate was hydrated. To this end, it was added to 3.4 L of milk (5 g/L) and stirred for 1 h at 4 °C. Then, the milk was heated at 90–95 °C for 5 min, under continuous stirring. After natural cooling, when the temperature reached 42 °C, the probiotic cultures were added (4.5 g L^−1^, according to manufacturer’s instructions). Similarly, the probiotic bacterial strains were added (0.34 g L^−1^, according to the manufacturer’s instructions). Next, 100 mL portions of the mixture were transferred into plastic polypropylene cups, of 250 mL capacity. The as-prepared dessert yogurts were then thermo-sealed with a multilayer aluminum foil and placed in an incubation chamber at a constant temperature of 42 °C for ca. 4–5 h. After complete solidification, samples were transferred to a home-type refrigerator at 4 ± 1 °C where they were stored for 21 days.

### 2.2. Semi-Quantitative Determination of Volatile Compounds

Semi-quantitative determination of volatile compounds was performed on day 21 of storage using the solid-phase microextraction technique (SPME) followed by gas chromatography coupled to a mass spectrometry detector (GC-MS). For this reason, an DVB/CAR/PDMS 50/30 μm (Divinylbenzene/Carboxen/Polydimethylsiloxane) SPME fiber purchased from Supelco, (Bellefonte, PA, USA) was used. The chromatographic system used for the analysis of the volatile components consisted of an Agilent 7890 A gas chromatograph coupled to Agilent 5975 C inert XL MSD mass spectrometer (Wilmington, DE, USA). The chromatographic column was DB-5MS, 60 m × 0.32 mm × 1 μm (J & W Scientific, Folsom, CA, USA).

For the determination of volatile compounds, a previously reported method was employed, with minor modifications [19]. In brief, 5.00 ± 0.01 g of dessert yogurt sample and 10 μL of internal standard (4-methyl-2-pentanone) were placed in a 20 mL glass vial. The vial was sealed with a PTFE/silicone septum and placed in a 45 °C water bath for 15 min to achieve equilibrium. The DVB/CAR/PDMS 50/30 μm (SPME) fiber was then exposed to the headspace area of the sample to extract the volatile compounds. After 10 min the fiber was placed in the Gas Mass Chromatograph-Spectrometer and thermal desorption of the volatile compounds took place. 

The optimized chromatographic conditions employed were: injector temperature: 260 °C, the split ratio: 2:1. For the separation of the compounds, the following oven temperature program was employed: initial temperature 40 °C for 5 min, then increased to 115 °C at a rate of 10 °C min^−1^, and further increased to 270 °C at a rate of 8 °C min^−1^ and held for 2 min. Finally, the mass transfer line temperature was 260 °C, the ion source temperature was set at 230 °C and the quadruple temperature was 150 °C. The mass acquisition range (*m*/*z*) was between 35 and 350. All measurements were carried out in triplicate. The identification of the volatile compounds was carried out using the National Institute of Standards Technology Mass Spectral Database library Wiley7-NIST 05 [17] and by determining the relevant retention indices (RI) of the volatile compounds. The RIs were calculated using a standard mixture of alkanes (C_8_–C_20_) (Fluka, Buchs, Switzerland) and analyzed under the same experimental conditions.

### 2.3. Organoleptic Evaluation

The organoleptic attributes of the prepared dessert yogurts were evaluated by a total of 51 untrained panelists (both faculty members and graduate students of the Laboratory of Food Chemistry, University of Ioannina), aged between 25 and 60, who regularly consume yogurt. The dessert yogurts were evaluated in terms of color, texture, odor and taste, using a 1–5 scoring scale with 5 corresponding to the most liked sample and 1 corresponding to the least liked sample. A score of 3 was taken as the lower limit of product acceptability. The evaluation took place in individual booths, under controlled conditions of light, temperature, and humidity. Each panelist tested samples (given only minimum information, regarding the addition of probiotic bacterial strains of chilled dessert yogurts (5 g) and rinsed their mouth with mineral water after assessing each sample.

### 2.4. Statistical Analysis

The experiment was repeated twice with three determinations per replicate (*n* = 2 × 3 = 6). Statistical analysis of data was performed using Analysis of Variance (ANOVA) followed by Duncan’s post hoc test. Statistically significant differences were considered for *p* < 0.05.

## 3. Results and Discussion

It has been reported in the literature [7,20] that the aroma and taste of yogurt are mainly due to the presence of nonvolatile and volatile carboxylic acids and carbonyl compounds; in particular, carbonyl compounds are believed to have a significant effect on the final aroma of yogurt due to their relatively high concentrations. Among the nonvolatile flavor compounds, lactic acid is a major contributor to yogurt flavor. Among the volatile compounds acetaldehyde, acetone, acetoin, and diacetyl in addition to acetic, formic, propanoic, butanoic, and hexanoic acids significantly contribute to yogurt aroma [6].

### 3.1. Volatile Compounds

The volatile flavor compounds of the dessert yogurts made from cow and goat milk, using three different cultures (sweet, classic, and acidic), with or without the addition of probiotic bacterial strains are presented in Table 1, Table 2, Table 3 and Table 4. These include aldehydes, alcohols, ketones, acids, hydrocarbons and terpenes. 

#### 3.1.1. Aldehydes

Aldehydes can be formed through either the degradation of milk fat (unsaturated fatty acids are oxidized to hydroperoxides, which in turn yield aldehydes through the action of hydroperoxidelase) or from amino acid catabolism [6,21]. However, aldehydes can also be oxidized to produce carboxylic acids or be reduced to yield alcohols via enzymatic reactions. Therefore, the final amount of aldehydes in yogurts is dependent on the activity of the enzymes present in microorganisms [21]. With regard to the aldehydes identified in cow dessert yogurts, without the addition of *Bifidobacterium* BB-12, only acetaldehyde was determined (54.87 ± 12.15 mg kg^−1^), in the sample prepared using the classic culture. In contrast, when *Bifidobacterium* BB-12 was added, acetaldehyde, pentanal, hexanal, and nonanal were determined in dessert yogurts (total aldehyde content: 30.36 ± 23.66 mg kg^−1^ for the classic and 12.86 ± 0.47 mg kg^−1^ for the acidic culture). The addition of *Bifidobacterium* BB-12 led to changes in the pH and composition of milk during fermentation (unlike dessert yogurts prepared without the addition of probiotic bacteria). The initial pH of cow milk was 6.70 and for goat milk it was 6.65. On day 21, The pH of cow dessert yogurt without *Bifidobacterium BB-12* was: 4.36 for the mild culture, 4.28 for the classic culture, 4.23 for the acidic culture and with *Bifidobacterium BB-12*: 4.31 for the mild culture, 4.22 for the classic culture and 4.16 for the acidic culture. These pH changes caused the activation of enzymes in *Bifidobacterium* BB-12, resulting in their combination with oxidation in the production of other aldehydes, in addition to acetaldehyde [8,9].

Acetaldehyde is one of the most important compounds in fermented dairy products, as it contributes a light, fresh, green, and strong odor [6,10]. Generally, it has been suggested that acetaldehyde may result from the degradation of various compounds such as fatty acids, glucose, catechol, glyceraldehydes, and amino acids such as threonine and glycine [11]. However, the most important pathway of acetaldehyde formation is reported to be the breakdown of threonine into acetaldehyde and glycine, which is catalyzed by the enzyme threonine aldolase [12]. This enzyme is present in both the *Streptococcus thermophilus* and *Lactobacillus bulgaricus* microorganisms, but the production of acetaldehyde by the two microorganisms is not identical. Bacterial cultures that allow acetaldehyde to form without particularly acidifying the yogurt are more desirable [13]. Acetaldehyde was mainly identified in larger amounts in the cow milk; the dessert yogurt prepared using the classic culture was found to be consistent with previous studies in which the classic culture produces a more desirable aroma in the dessert yogurt due to acetaldehyde [14].

However, the above-mentioned trend was not observed in the dessert yogurts from goat milk. Both with the addition or in the absence of *Lactobacillus acidophilus* LA-5, no aldehydes other than acetaldehyde were determined (7.12 ± 4.08 mg kg^−1^ for the mild and 66.09 ± 18.12 mg kg^−1^ for the classic culture in the absence of *Lactobacillus acidophilus* LA-5 and 54.50 ± 1.00 mg kg^−1^ for the mild, 11.13 ± 0.04 mg kg^−1^ for the classic and 17.06 ± 4.35 mg kg^−1^ for the acidic culture in the presence of *Lactobacillus acidophilus* LA-5). This may be due to the fact that different probiotic bacterial strains were used to prepare dessert yogurts from cow and goat milk. Similarly, differences in the amount of acetaldehyde in goat dessert yogurts with the addition of *Lactobacillus acidophilus* LA-5 compared to those prepared without *Lactobacillus acidophilus* LA-5 may be due to the specific enzymatic activity of *Lactobacillus acidophilus* LA-5 bacteria [22,23]. On day 21 of storage, the pH values for the goat dessert yogurt without *Lactobacillus acidophilus* LA-5 were: 4.29 for the mild culture, 4.26 for the classic culture, 4.24 for the acidic culture and with *Lactobacillus acidophilus* LA5: 4.24 for the mild culture, 4.21 for the classic culture and 4.12 for the acidic culture. The lower pH values observed with the addition of *Lactobacillus acidiphilus* LA-5, compared to the addition of *Bifidobacterium* BB-12, confirms the stronger acidifying effect of the former bacterial strain.

According to Sandine et al. [24], good yogurt flavor is produced when 8.0 ppm or more of acetaldehyde is produced. In the present study, the acetaldehyde concentrations recorded were, in most cases, quite high, ranging from 6.60 ± 0.12 to 66.09 ± 18.12 mg kg^−1^. The lack of alcohol dehydrogenase enzyme in the bacteria, which is responsible for the conversion of acetaldehyde into ethanol, is suggested to be the reason behind the high acetaldehyde content [12]. As a result, ethanol, a documented volatile compound of yogurt [6,7] was not determined in any of the dessert yogurts in the present study. According to the literature [25] yogurt products with a very low acetaldehyde content still have a typical yogurt aroma, suggesting that acetaldehyde is only one component of yogurt aroma and does not account for the overall yogurt aroma. Pentanal, hexanal and octanal and nonanal have also been reported as components of the volatile fraction of yogurt [6] formed during the oxidation of milk fat. Of these, pentanal, hexanal and nonanal were determined in the present study in cow yogurt with the addition of *Bifidobacterium* BB-12. The presence of such aldehydes in the probiotic yogurt prepared with *Bifidobacterium* BB-12 may be associated with its relatively low acceptability by consumers (see organoleptic evaluation).

#### 3.1.2. Ketones

Ketones are a major class of volatile compounds identified in yogurt. They are derived both from raw milk (and as such, dessert yogurts from cow milk are expected to differ from those from goat milk in their ketone content) and from the processing parameters of yogurt production, due to the β-oxidation metabolic pathway of unsaturated fatty acids [15,26]. Dessert yogurts made from cow milk, prepared with the mild culture, without the addition of Bifidobacterium BB-12, had had a ketones content of around three times lower than the corresponding dessert yogurts prepared with the classic and acidic cultures (52.52 ± 12.98 mg kg^−1^ for the mild, 160.90 ± 52.47 mg kg^−1^ for the classic and 147.60 ± 50.54 mg kg^−1^ for the acidic culture), probably due to the specific action of the mild culture. The same difference in the content of ketones was observed between the dessert yogurts from cow milk with different cultures, to which Bifidobacterium BB-12 were added (68.39 ± 39.45 mg kg^−1^ for the mild, 143.46 ± 23.83 mg kg^−1^ for the classic and 202.52 ± 5.98 mg kg^−1^ for the acidic culture). However, for a given culture, no significant differences were observed in the ketone content between yogurts with or without Bifidobacterium BB-12, which could be associated with the specific culture used and not with the addition of Bifidobacterium BB-12.

In the case of dessert yogurts from goat milk without *Lactobacillus acidophilus* LA-5, the same rough tendency was observed in the content of ketones. That is, dessert yogurts with the mild culture had a significantly lower content of ketones than those prepared with the classic and acidic culture (128.72 ± 69.16 mg kg^−1^ for the mild, 184.22 ± 26.41 mg kg^−1^ for the classic and 187.93 ± 77.79 mg kg^−1^ for the acidic culture). However, the case of dessert yogurts from goat milk to which *Lactobacillus acidophilus* LA-5 was added was quite different. In this case, the dessert yogurts with the mild culture had ca. a 2.6 times higher content of ketones, compared to the corresponding dessert yogurts to which no *Lactobacillus acidophilus* LA-5 were added (338.34 ± 128.24 mg kg^−1^ vs. 128.72 ± 69.16 mg kg^−1^). This value was ca. 4 times higher than that of ketones in dessert yogurts with *Lactobacillus acidophilus* LA-5, which were prepared using the classic and acidic cultures (338.34 ± 128.24 mg kg^−1^ vs. 87.85 ± 18.30 and 74.04 ± 10.97 mg kg^−1^). These two dessert yogurts showed ca. 50% reduction in the content of their ketones, compared to desserts prepared without *Lactobacillus acidophilus* LA-5 (87.85 ± 18.30 vs. 184.22 ± 26.41 mg kg^−1^ for the classic and 74.04 ± 10.97 vs. 187.93 ± 77.79 mg kg^−1^ for the acidic culture). This finding is consistent with results previously reported in the literature, as it is reported that the addition of bacterial strains that grow easily in milk, such as *Lactobacillus acidophilus*, affects the formation of ketones [27]. Therefore, in the case of dessert yogurts derived from cow milk, the addition of *Bifidobacterium* BB-12 does not seem to significantly affect their ketone content and as such, their odor. In contrast, in dessert yogurts from goat milk, the addition of *Lactobacillus acidophilus* LA-5 significantly increased the ketone content when the mild culture was used while decreasing the ketone content when the other two cultures were used.

Among the determined ketones, diacetyl (2,3-butanedione) is an important aroma compound, contributing to yogurt aroma with buttery notes. It is formed through the fermentation of citrate present in milk. Typical concentrations of diacetyl in yogurt range from 0.2–3 mg kg^−1^ [28]. In the present study, substantially higher concentrations of diacetyl were recorded. Acetoin (3-hydroxy-2-butanone) is a common flavor constituent in many fermented dairy products that is readily converted from diacetyl [6]. It has a mild creamy, butter-like flavor, similar to that of diacetyl but considerably weaker. Acetoin is readily formed from diacetyl by the enzyme diacetyl reductase. Typical acetoin concentrations in yogurt range from 1.2 to 28 mg kg^−1^ [28]. In the present work, significantly higher concentrations of acetoin were recorded. Acetone (2-propanone) originates either from milk or from the yogurt bacterial cultures and is of minor importance to flavor contribution in dairy products [29]. It has a sweet fruity aroma and contributes positively to the flavor of yogurt. The typical acetone content in yogurt varies from 0.3 to 4 mg kg^−1^ [27,28]. In the present study, significantly higher concentrations of acetone were recorded.

In general, the presence of Bifidobacteria has not been reported to affect the production of ketones by the microorganisms of the starter culture, whereas, on the contrary, the probiotic microorganism *Lactobacillus acidophilus* affects the production of ketones [27]. This can be due to the increased rate of their citrate metabolism [30]. This was validated from our findings, since the cow dessert yogurts that were fortified with Bifidobacteria showed no increase in their flavor, compared to goat dessert yogurts that contained *Lactobacillus acidophilus* and exhibited an improvement in flavor (Section 3.2). Beshkova et al. [31] reported concentrations of 14.1 to 17.3 mg kg^−1^ for acetaldehyde; 1.6 to 2.0 mg kg^−1^ for diacetyl; 1.7 to 2.2 mg kg^−1^ for acetoin and 0.66 to 0.75 mg kg^−1^ for acetone in Bulgarian yogurts. 2-pentanone, 2-heptanone and 2-nonanone have also been recorded in the flavor profile of yogurts [6]. Imhof et al., [32,33] determined the key aroma components in Switzerland yogurts and identified six volatiles (acetaldehyde, dimethylsulphide, diacetyl, 2,3-pentanedione, l-limonene, and undecanal) as having a high impact on yogurt flavor. These researchers noted that the obtained results should be interpreted with caution because the main ingredients (fat, proteins, and carbohydrates) in yogurt can significantly reduce the actual release of volatiles. Other carbonyl compounds including 2,3-pentanedione were found to contribute to the aroma of yogurt [32]. Specifically, 2,3-pentanedione is an impact flavor compound of yoghurt [34] and may be formed from a-aceto-a-hydroxybutyrate, an intermediate of isoleucine metabolism [33] 2,3-pentanedione was also determined in the flavor profile of all yogurt samples in the present study.

#### 3.1.3. Volatile Acids

Both non-volatile and volatile carboxylic acids are important flavor compounds in dairy products, as they enhance product sensory properties [35]. Even though lactic acid is not a volatile carboxylic acid, it is included in the present discussion due to its crucial importance in yogurt flavor. Lactic acid is the prime source of flavor in fermented dairy products, responsible for the refreshing sour/acidic flavor of yogurt [36]. During fermentation, a good portion (20–40%) of lactose is transformed into lactic acid. Acidity is a key factor in yogurt flavor, producing a pH of around 4.4–4.2. Ott et al. [37] showed that there are important flavor differences between traditional acidic and mild, less acidic yogurts, which are mainly due to the differences in acidity and not due to different concentrations of the three flavor impact compounds (acetaldehyde, diacetyl, and 2,3-pentanedione). These authors considered lactic acid as the most important component of yogurt flavor. Carboxylic acids usually derive from lipolysis, proteolysis or lactose metabolism [15,38].

Regarding the volatile acid content of the yogurt samples, it was observed that the different samples contained different carboxylic acids. More specifically, in dessert yogurts made from cow milk without the addition of Bifidobacterium BB-12, hexanoic acid (contributing with pungent, rancid, flowery notes) was determined in small amounts in the case of the mild and classic cultures, while in the case of the acidic culture, no volatile acids were identified. When Bifidobacterium BB-12 was added, acetic acid was determined in particularly high concentrations (120 to 151 mg kg^−1^), except in the case of the classic culture. In a previous study, it was reported that the main difference in volatiles between cow milk either unfermented or fermented with Bifidibacterium BB-12, was the high concentration of acetic acid produced in the latter [9]. Despite the weak ability of bifidobacteria to ferment lactose, the acetic acid content in fermented milk confirmed their heterofermentative nature.

Acetic acid imparts a vinegar flavor to yogurt; therefore, when found at high concentrations, it can render the dessert yogurt unacceptable by consumers. Therefore, these samples are expected to show inferior organoleptic properties compared to yogurt desserts from cow milk, to which no Bifidobacterium BB-12 are added. This was validated, as can be seen from the organoleptic evaluation results in Section 3.2. Alonso and Fraga [39] reported an acetic acid concentration ranging from 0.5 to 18.8 mg kg^−1^ in commercial yogurts.

In the case of dessert yogurts from goat milk without the addition of *Lactobacillus acidophilus* LA-5, no volatile acids were identified, while in dessert yogurts prepared with the addition of *Lactobacillus acidophilus* LA-5, only butanoic acid was identified. Butanoic acid imparts a cheesy flavor, and therefore the corresponding samples are expected to have improved organoleptic properties [38]. This was validated, as can be seen from the organoleptic evaluation results in Section 3.2.

The thermophilic Streptococcus and Lactobacillus cultures are known to produce acetic, butyric, and caproic (hexanoic) acids [31].

#### 3.1.4. Hydrocarbons

Hydrocarbons are primarily by-products of the lipid oxidation of raw milk. Although identified in almost all samples, hydrocarbons do not contribute to yogurt aroma due to their low concentrations and high odor threshold [40].

Hydrocarbons were determined in all samples (3.51–16.47 mg kg^−1^ in the cases of cow dessert yogurts without the addition of *Bifidobacterium* BB-12, and 12.58–19.89 mg kg^−1^ in the cases with the addition of *Bifidobacterium* BB-12. The respective hydrocarbon content ranged between 12.01–40.03 mg kg^−1^ in goat dessert yogurts with the addition of *Lactobacillus acidophilus* LA-5), while no hydrocarbons were determined in dessert yogurts from goat milk, to which no *Lactobacillus acidophilus* LA-5 were added. The carbohydrate content of dessert yogurts from cow milk with the addition of *Bifidobacterium* BB-12 was generally higher than that without the addition of *Bifidobacterium* BB-12, while the corresponding dessert yogurts from goat milk had a considerably higher content of hydrocarbons. In general, it has been reported that different hydrocarbons in different amounts are produced when either *Streptococcus thermophilus* or *Lactobacillus bulgaricus* are added individually to milk or when they are used in combination in the starter culture for fermentation [41]. Therefore, the observed differences in carbohydrate content and their amount among the dessert yogurts may be attributed to the use of different starter cultures, but also to specific probiotics used as is the case of *Lactobacillus acidophilus* LA-5. According to the literature, the presence of specific hydrocarbons observed in dessert yogurts may also be due to various parameters during the milk transport and storage process [42].

#### 3.1.5. Terpenes

Regarding the terpene content of the samples, it was observed that in all dessert yogurts from cow milk (with or without the addition of *Bifidobacterium* BB-12) specific terpenes (limonene, α-pinene, caryophyllene) were determined in small amounts (<10 mg kg^−1^). In contrast, in yogurt desserts from goat milk, a drastically higher content of terpenes (37.03–63.08 mg kg^−1^) for samples without *Lactobacillus acidophilus* LA-5 and (74.04–338.34 mg kg^−1^) for samples with *Lactobacillus acidophilus* LA-5 was observed. Terpenes are natural plant secondary metabolites found in bushes, grass, and trees and can be found in milk. As a consequence, they can be found also in the dessert yogurts when the animals feed more on plants that are rich in terpenes [43,44]. The highest content of terpenes in dessert yogurts from goat milk is due to animal nutrition, according to previous reports [45,46].

### 3.2. Organoleptic Evaluation

**Figure 1:**
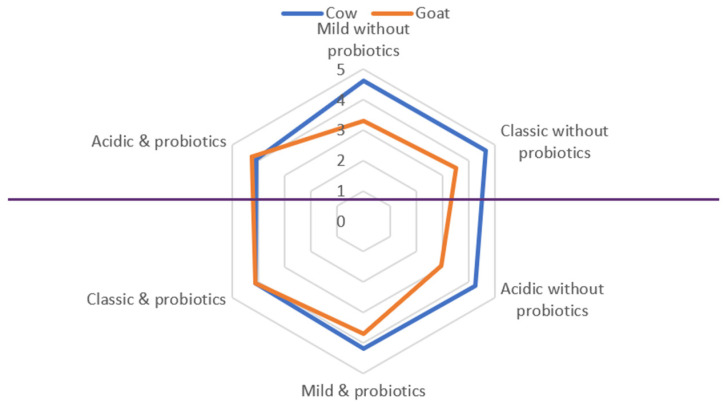
Spider graph of the yogurt dessert flavor.

The organoleptic evaluation showed that color and texture did not differ significantly among treatments (data not shown). Flavor (taste + odor) proved to be the most sensitive attribute in the evaluation of dessert yogurt quality. Table 5 shows flavor scores for both cow and goat dessert yogurts. Flavor scores for cow dessert yogurts ranged from 4.25 ± 0.13 to 4.65 ± 0.14 for the three cultures without the addition of *Bifidobacterium* BB-12. Respective taste scores for samples with *Bifidobacterium* BB-12 ranged from 4.06 ± 0.15 and 4.20 ± 0.10. The flavor of goat dessert yogurts ranged from 2.96 ± 0.25 to 3.50 ± 0.19 for the three cultures without the addition of *Lactobacillus acidophilus*. Respective flavor scores for samples with *Lactobacillus acidophilus* LA-5 ranged from 3.70 ± 0.15 to 4.25 ± 0.20. Generally, it can be observed that cow dessert yogurts, without *Bifidobacterium* BB-12 exhibited better flavor (*p* < 0.05) compared to those with *Bifidobacterium* BB-12. In contrast, significantly higher (*p* < 0.05) flavor scores were recorded for goat dessert yogurts with *Lactobacillus acidophilus* LA-5 compared to those without *Lactobacillus acidophilus* LA-5. Between the cow dessert yogurts without *Bifidobacterium* BB-12 and the goat dessert yogurts with *Lactobacillus acidophilus* LA-5, the former were more acceptable (*p* < 0.05) by the panelists. Therefore, it can be concluded that the different types of milk, the specific starter cultures used, and the addition of probiotic bacterial strains has a considerable impact on the flavor of dessert yogurts. Within the cow dessert yogurts without *Bifidobacterium* BB-12, the mild and classic starter culture recorded the highest flavor scores while within the goat dessert yogurts with *Lactobacillus acidophilus* LA-5, the classic and acidic starter culture recorded the highest flavor scores. The organoleptic evaluation results in the form of a spider graph are shown in Figure 1. As can be seen, the optimum results for cow milk dessert yogurts were recorded in the cases of the mild and classic starter cultures without the addition of *Bifidobacterium* BB-12, whereas in the case of goat milk dessert, the yogurt’s optimum results were recorded in case of the classic and acidic starter cultures with the addition of *Lactobacillus acidophilus* LA-5.

In a study by Ott et al. [34], during the characterization of the sensory properties of traditional acidic and mild, less-acidic yogurts by a trained panel using a descriptive approach, it was observed that the important flavor differences found between the two samples of yogurt were mainly due to the differences in the acidity and not due to different concentrations of the three aroma compounds (acetaldehyde, 2,3-butanedione, and 2,3-pentanedione). This observation emphasizes the importance of acidity in yogurt flavor in relation to the contribution of various volatile compounds to the overall flavor of yogurt. Finally, Costa et al., 2014 [18], when working with goat yogurt to which *Lactobacillus acidophilus* LA-5 had been added, reported an improvement in goat yogurt acceptability with the addition of the probiotic.

### 3.3. Correlation of Flavor Volatile Compounds to Organoleptic Evaluation Data

Pearson’s correlation of instrumental volatiles to organoleptic evaluation data are shown in Table 6.

Table 6 shows that: (1) organoleptic flavor correlates positively to aldehydes, ketones and to a lesser extent to carboxylic acids for cow dessert yogurt without the addition of *Bifidibacterium* BB-12; (2) the organoleptic flavor correlates positively to ketones and negatively to aldehydes and carboxylic acids for cow dessert yogurt with the addition of *Bifidobacterium* BB-12; (3) the organoleptic flavor correlates positively to aldehydes and ketones for the goat dessert yogurt without the addition of *Lactobacillus acidophilus* LA-5 and (4) the organoleptic flavor correlates highly positively to aldehydes and ketones for goat dessert yogurt with the addition of *Lactobacillus acidophilus* LA-5.

## 4. Conclusions

In this work, the effect of milk type, starter culture and the use of probiotic bacterial strains was investigated with respect to the resulting volatile compounds profile and organoleptic evaluation of cow and goat dessert yogurts. Among the different samples prepared, variations in the content of dessert yogurts in aldehydes, alcohols, ketones, volatile acids, hydrocarbons, and terpenes were recorded. From the obtained results, it can be concluded that dessert yogurts prepared from cow milk using the mild and classic starter culture and without the probiotic *Bifidobacterium* BB-12 and dessert yogurts prepared from goat milk using the classic and acidic starter culture with the probiotic *Lactobacillus acidophilus* LA-5 were sensorially more acceptable. This was primarily the result of the extent of product acidity (low in the use of mild and classic starter cultures without the addition of *Bifidobacterium* BB-12 in cow yogurts and the acidifying effect of probiotic bacterial strains *Lactobacillus acidophilus* LA-5 in goat yogurts) and secondarily of the variation of specific volatile compounds determined in yogurts, in line with the findings of Ott et al., 2000 [34]. Therefore, these combinations seem to be more promising for the development of commercial dessert yogurts.

## Figures and Tables

**Table 1:** Volatile compounds of cow dessert yogurts (mg kg^−1^), prepared with different cultures without the addition of *Bifidobacterium animalis* ssp. *lactis* BB-12.

Compound	RI_exp_	RI_lit_	Mild	Classic	Acidic
***Aldehydes***					
acetaldehyde	-	-	-	54.87 ± 12.15 ^a^	-
***Ketones***					
2-Propanone (acetone)	-	500	-	37.75 ± 18.88 ^a^	-
2,3-Butanedione (Diacetyl)	566	584	14.02 ± 0.22 ^a^	43.89 ± 7.21 ^cd^	36.39 ± 10.35 ^bc^
2-Pentanone	681	689	-	6.42 ± 0.88 ^b^	-
2,3-Pentanedione	693	700	-	-	16.47 ± 1.02 ^c^
3-Hydroxy-2-butanone(Acetoin)	720	721	30.24 ± 10.95 ^a^	63.26 ± 13.07 ^a^	73.72 ± 32.27 ^ab^
2-Heptanone	891	899	6.82 ± 1.40 ^a^	9.58 ± 2.43 ^a^	15.69 ± 4.84 ^a^
Dihydro-2-methyl-3 (2H)-thiophenone	1005	994	-	-	2.62 ± 1.31 ^a^
2-Nonanone	1093	1095	1.45 ± 0.43 ^a^	-	2.71 ± 0.75 ^b^
**Total**			52.52 ± 12.98	160.90 ± 52.47	147.6 ± 50.54
***Carboxylic Acids***					
Hexanoic acid(Caproic acid)	923	934	2.40 ± 1.39 ^a^	3.20 ± 1.85 ^a^	-
***Hydrocarbons***					
Toluene	774	771	-	-	4.29 ± 1.75 ^b^
2.4-Dimethyl-1-heptane	821	820	2.58 ± 1.29 ^a^	-	-
1-Decene	992	990	-	-	3.44 ± 0.10 ^c^
Decane	1000	1000	3.06 ± 0.65 ^a^	3.51 ± 1.03 ^a^	5.48 ± 3.68 ^a^
Dodecane	1193	1199	-	-	3.27 ± 1.90 ^a^
**Total**			5.64 ± 1.94	3.51 ± 1.03	16.47 ± 7.33
***Terpenes***					
Limonene	1044	1039	-	4.54 ± 0.65 ^b^	2.69 ± 2.10 ^ab^
Caryophyllene	1465	1451	-	1.93 ± 1.19 ^a^	-
**Total**				6,47 ± 1.89 ^a^	2.69 ± 2.10
***Total volatiles***					
			66.21 ± 18.25	177.59 ± 58.22	183.24 ± 67.4

^a,b,c,d^ Means with different letters in the same row are statistically different (*p* < 0.05); RI_exp_: Experimental retention index; RI_lit_: Retention index based on literature [17].

**Table 2:** Volatile compounds of cow dessert yogurts (mg kg^−1^), prepared with different cultures with the addition of *Bifidobacterium animalis* ssp. *lactis* BB-12.

Compound	RI_exp_	RI_lit_	Mild	Classic	Acidic
***Aldehydes***					
Acetaldehyde			-	21.59 ± 15.97 ^b^	6.60 ± 0.12 ^ab^
Pentanal	697	695	-	4.75 ± 3.36 ^a^	4.53 ± 0.1 ^a^
Hexanal	801	798	-	5.41 ± 2.95 ^b^	-
Nonanal	1108	1105	-	-	1.73 ± 0.25 ^a^
**Total**			-	30.36 ± 23.66	12.86 ± 0.47
***Alcohols***					
1,8-Cineole	1044	1038	1.81 ± 1.01 ^a^	-	-
***Ketones***					
2-Propanone	-	500	-	28.84 ± 0.52 ^b^	10.52 ± 0.10 ^ab^
2,3 Butanedione	566	584	21.28 ± 12.16 ^ab^	30.20 ± 0.15 ^abc^	55.11 ± 1.06 ^d^
2-Pentanone	681	689	-	6.11 ± 0.71 ^b^	-
2,3-Pentanedione	693	700	-	8.39 ± 0.58 ^b^	-
3-Hydroxy-2-butanone	720	721	39.25 ± 22.96 ^a^	58.03 ± 16.99 ^a^	120.42 ± 4.67 ^b^
2-Heptanone	891	899	7.86 ± 4.33 ^a^	11.89 ± 4.88 ^a^	13.57 ± 0.13 ^a^
2-Nonanone	1093	1095	-	-	2.57 ± 0.02 ^b^
**Total**			68.39 ± 39.45	143.46 ± 23.83	202.52± 59.8
***Carboxylic Acids***					
Acetic acid	923	934	151.46 ± 77.25	-	119.94 ± 84.81
***Hydrocarbons***					
1-Decene	992	990	1.91 ± 0.51 ^b^	1.94 ± 0.65 ^b^	3.27 ± 0.65 ^c^
Nonanene	900	900	3.55 ± 0.63 ^b^	4.88 ± 0.12 ^b^	5.12 ± 0.83 ^a^
Toluene	774	771	2.12 ± 1.22 ^a^	-	-
Decane	1000	1000	5.15 ± 1.29 ^a^	5.76 ± 0.86 ^a^	7.42 ± 1.33 ^a^
1-Methyl-2-(1-methylethyl)-benzene	1039	1041	1.15 ± 0.79 ^a^	-	-
Dodecane	1193	1199	2.52 ± 0.70 ^b^	-	4.08 ± 1.63 ^ab^
Total			16.41 ± 5.12	12.58 ± 1.63	19.89 ± 4.41
***Terpenes***					
α-Pinene	936	943	-	1.50 ± 0.43 ^a^	-
Limonene	1044	1039	3.01 ± 2.71 ^ab^	4.15 ± 1.31 ^b^	3.12 ± 0.94 ^ab^
Total			3.01 ± 2.71	5.65 ± 1.74	3.12 ± 0.94
***Total volatiles***					
			271.44 ± 125.54	192.05 ± 50.86	358.33 ± 96.61

^a,b,c,d^ Means with different letters in the same row are statistically different (*p* < 0.05); RI_exp_: Experimental retention index; RI_lit_: Retention index based on literature [17].

**Table 3:** Volatile compounds of goat dessert yogurts (mg kg^−1^), prepared with different cultures, without the addition of *Lactobacillus acidophilus* LA-5.

Compound	RI_exp_	RI_lit_	Mild	Classic	Acidic
***Aldehydes***					
Acetaldehyde	-	-	7.12 ± 4.08 ^a^	66.09 ± 18.12 ^b^	-
***Ketones***					
2-Propanone	-	500	33.50 ± 24.73 ^a^	-	33.22 ± 11.10 ^a^
2,3 Butanedione	566	584	37.24 ± 19.76 ^a^	97.19 ± 18.72 ^ab^	116.37 ± 48.60 ^ab^
2-Pentanone	681	689	7.58 ± 1.09 ^c^	4.30 ± 0.10 ^b^	7.50 ± 1.40 ^c^
2,3-Pentanedione	693	700	20.42 ± 9.77 ^ab^	17.11 ± 4.59 ^ab^	16.78 ± 11.86 ^ab^
3-Hydroxy-2-butanone	720	721	13.82 ± 6.34 ^ab^	32.16 ± 1.00 ^ab^	-
2-Heptanone	891	899	16.16 ± 7.47 ^c^	17.33 ± 1.00 ^c^	14.06 ± 4.83 ^bc^
Dihydro-2-methyl-3 (2H)-thiophenone	1005	994	-	16.14 ± 1.00 ^a^	-
**Total**			128.72 ± 69.16	184.22 ± 26.41	187.93 ± 77.79
***Terpenes***					
α-Pinene	936	943	48.83 ± 21.77 ^b^	23.54 ± 5.47 ^ab^	32.85 ± 12.35 ^ab^
Camphene	969	950	-	13.49 ± 2.14 ^bc^	15.67 ± 5.91 ^c^
β-Pinene	936	978	6.69 ± 2.82 ^a^	-	-
Limonene	1044	1039	7.56 ± 6.24 ^a^	-	-
**Total**			63.08 ± 30.83	37.03 ± 7.61	48.52 ± 18.26
***Total volatiles***					
			198.92 ± 104.07	287.34 ± 52.14	236.45 ± 96.05

^a,b,c^ Means with different letters in the same row are statistically different (*p* < 0.05); RI_exp_: Experimental retention index; RI_lit_: Retention index based on literature [17].

**Table 4:** Volatile compounds of goat dessert yogurts (mg kg^−1^), prepared with different cultures with the addition of *Lactobacillus acidophilus* LA-5.

Compound	RI_exp_	RI_lit_	Mild	Classic	Acidic
***Aldehydes***					
Acetaldehyde	-	-	54.50 ± 1.00 ^b^	11.13 ± 0.04 ^a^	17.06 ± 4.35 ^a^
***Ketones***					
2-Propanone	-	500	-	15.01 ± 2.76 ^a^	-
2,3 Butanedione	566	584	153.79 ± 82.62 ^b^	30.90 ± 5.11 ^a^	55.76 ± 1.00 ^ab^
2-Pentanone	681	689	-	5.91 ± 0.62 ^b^	-
2,3-Pentanedione	693	700	43.67 ± 23.67 ^b^	4.98 ± 0.30 ^a^	12.68 ± 8.97 ^ab^
3-Hydroxy-2-butanone	720	721	140.88 ± 21.97 ^ab^	31.05 ± 9.51 ^ab^	0
2-Heptanone	891	899	-	-	5.30 ± 1.00 ^ab^
**Total**			338.34 ± 128.24	87.85 ± 18.30	74.04 ± 10.97
***Carboxylic Acids***					
Butanoic acid(Butyric acid)	721	735	-	5.63 ± 0.15 ^a^	-
***Hydrocarbons***					
Cyclopentane	563	564	37.66 ± 26.63 ^b^	4.77 ± 1.62 ^a^	-
2,2,4 Trimethyl-pentane	684	668	-	-	15.10 ± 4.83 ^a^
2,4 Dimethyl-heptane	697	700	-	-	4.31 ± 0.53 ^a^
4-Methyl-octane	800	800	-	-	3.01 ± 0.1 ^a^
Nonane	900	900	-	3.87 ± 0.1 ^a^	4.23 ± 1.01 ^a^
1,3 Dimethyl-benzene	904	900	-	-	5.61 ± 1.30 ^a^
2,2,4,6,6 Pentamethyl-Heptane	997	997	-	-	3.68 ± 0.09 ^a^
Decane	999	1000	-	3.37 ± 0.1 ^a^	4.09 ± 1.25 ^a^
**Total**			37.66 ± 26.63	12.01 ± 1.72	40.03 ± 9.02
***Terpenes***					
α-Pinene	936	943	40.59 ± 24.10 ^ab^	4.30 ± 0.60 ^a^	6.56 ± 0.32 ^a^
Camphene	969	950	16.90 ± 8.59 ^c^	-	-
**Total**			57.49 ± 32.69	4.30 ± 0.60	6.56 ± 0.32
***Total volatiles***					
			487.99 ± 188.77	120.92 ± 20.81	137.69 ± 24.66

^a,b,c^ Means with different letters in the same row are statistically different (*p* < 0.05); RI_exp_: Experimental retention index; RI_lit_: Retention index based on literature [17].

**Table 5:** Mean values and standard deviations of flavor of dessert yogurts (after 1 day of storage) made using different milk types, starter cultures and probiotic bacterial strains.

	Mild without Probiotics	Classic without Probiotics	Acidic without Probiotics	Mild & Probiotics	Classic & Probiotics	Acidic & Probiotics
**Cow**	4.61 ± 0.18 ^e^	4.65 ± 0.14 ^e^	4.25 ± 0.13 ^d^	4.20 ± 0.10 ^d^	4.11 ± 0.11 ^d^	4.06 ± 0.15 ^d^
**Goat**	3.31 ± 0.22 ^b^	3.50 ± 0.19 ^bc^	2.96 ± 0.25 ^a^	3.70 ± 0.15 ^c^	4.11 ± 0.16 ^d^	4.25 ± 0.20 ^d^

^a,b,c,d,e^ Means with different letters in the same row are statistically different (*p* < 0.05).

**Table 6:** Correlation of dessert yogurt volatile compounds to organoleptic evaluation results.

	**Cow Dessert Yogurt without the Addition of *Bifidobacterium* BB-12.**							
	**Aldehydes**		**Ketones**	**Carboxylic Acids**	**Hydrocarbons**	**Terpenes**	**Total Volatiles**	**Flavor**
Aldehydes	1							
Ketones	0.560		1					
Carboxylic Acids	0.636		0.141	1				
Hydrocarbons	−0.501		0.423	−0.568	1			
Terpenes	0.459 **		0.491 **	0.382	0.006	1		
Total volatiles	0.440		0.990 **	0.052	0.548	0.125	1	
Flavor	0.496 *		0.571 *	0.284*	−0.584	0.230	−0.146	1
	**Cow Dessert Yogurt with the Addition of *Bifidobacterium* BB-12.**							
	**Aldehydes**	**Alcohols**	**Ketones**	**Carboxylic Acids**	**Hydrocarbons**	**Terpenes**	**Total Volatiles**	**Flavor**
Aldehydes	1							
Alcohols	−0.532	1						
Ketones	0.461	−0.574	1					
Carboxylic Acids	−0.551	0.654	−0.029	1				
Hydrocarbons	−0.177	0.282	0.488	0.854 **	1			
Terpenes	0.696 *	0.028	0.35	−0.084	0.169	1		
Total volatiles	−0.109	0.263	0.527	0.822 **	0.997 **	0.224	1	
Flavor	−0.157 *	0.511	0.342*	−0.370 *	0.184	0.230	0.180	1
	**Goat Dessert Yogurt without the Addition of *Lactobacillus acidophilus* LA-5.**							
	**Aldehydes**		**Ketones**		**Terpenes**		**Total Volatiles**	**Flavor**
Aldehydes	1							
Ketones	0.259		1					
Terpenes	−0.295		0.500		1			
Total volatiles	0.498		0.946 **		0.500		1	
Flavor	0.672 *		0.485 *		0.128		0.426	1
	**Goat Dessert Yogurt with the Addition of *Lactobacillus acidophilus* LA-5.**							
	**Aldehydes**		**Ketones**	**Carboxylic Acids**	**Hydrocarbons**	**Terpenes**	**Total Volatiles**	**Flavor**
Aldehydes	1							
Ketones	0.889 **		1					
Carboxylic Acids	0.601		0.408	1				
Hydrocarbons	0.414		0.556	−0.687 *	1			
Terpenes	0.852 **		0.991 **	−0.450	0.638	1		
Total volatiles	0.892 **		0.996 **	−0.470	0.619	0.995 **	1	
Flavor	0.772 *		0.677 *	0.237	0.017	−0.626	−0.648	1

** Correlation is significant at the 0.01 level, * Correlation is significant at the 0.05 level.

## Data Availability

The data presented in this study are available on request from the corresponding author.
